# Sugar-Sweetened Beverage Intake and Motor Function Among Autistic and Typically Developed Children

**DOI:** 10.3389/fnut.2022.905025

**Published:** 2022-07-14

**Authors:** Muqing Cao, Tingfeng Gu, Chengkai Jin, Xiuhong Li, Jin Jing

**Affiliations:** Department of Maternal and Child Health, School of Public Health, Sun Yat-sen University, Guangzhou, China

**Keywords:** sugar-sweetened beverage, motor function, autism, physical activity, sedentary time

## Abstract

**Background and Objectives:**

The relationship between brain function and sugar-sweetened beverages (SSBs) is widely explored, but the motor function was not included. We aim to explore the relationship between SSBs and motor function among children with or without autism.

**Methods:**

Participants were a representative autism sample (ASD, *n* = 106) comprising ages ranging 6–9 years and their age-matched typical counterparts (TD, *n* = 207), recruited in the research center of Guangzhou, China. Valid questionnaires of parent-reported including weekly SSBs intake, physical activity (PA), sedentary time (ST), and motor coordination function was used to collect relevant information. SSBs intake was further classified as no intake (no habit of taking SSBs), small to medium intake (<375 ml/week), and large intake (375 ml/week or more). Physical activity, sedentary time, and motor coordination function among the mentioned three groups as well as ASD *vs*. TD was compared *via* general linear models.

**Results:**

Compared with TD children, ASD children showed less vigorous PA (4.23 ± 0.34 h *vs*. 2.77 ± 0.49 h, *p* = 0.015) as well as overall sedentary time (5.52 ± 1.89 h *vs*. 3.67 ± 0.28 h, 3.49 ± 0.16 h *vs*. 2.68 ± 0.24 h, and 34.59 ± 1.15 h *vs*. 23.69 ± 1.69 h, TD *vs*. ASD, sedentary time at weekdays, weekends and total ST in a week, respectively, all *p* < 0.05), lower scores in the developmental coordination disorder questionnaire (fine motor and handwriting: 14.21 ± 0.26 *vs*. 12.30 ± 0.38, general coordination: 28.90 ± 0.36 *vs*. 25.17 ± 0.53, control during movement: 24.56 ± 0.36 *vs*. 18.86 ± 0.53, and total score: 67.67 ± 0.75 *vs*. 56.33 ± 1.10, TD *vs*. ASD, all *p* < 0.05). Stratified by SSBs intake, TD children with small to medium SSBs intake showed the lowest sedentary time both on weekdays and weekends (all *p* < 0.05), they also performed worst in fine motor and handwriting skills (*p* < 0.05).

**Conclusion:**

The association between SSBs and motor function was observed in typical development children, but not autistic children. A larger sample size study with a longitudinal design is warranted to confirm the association between SSBs and sedentary time among typically developed children and the potential causation direction.

## Introduction

The intake of sugar-sweetened beverages (SSBs) is well explored in regard to the metabolic status of children (1). According to reports published in recent years, SSBs are also associated with brain function (2). For instance, both research conducted on humans and rodents (3, 4) showed a negative impact of excessive sugar intake on the brain, especially on executive (5) and memory function (6), which indicated a potentially negative effect on overall brain function.

However, motor function, as an important domain of brain function, has been neglected in the research pertaining to SSBs and the brain. Motor function is the basis of physical activity and fitness in children; thus, it plays a vital role in the long-term health and well-being of children. The current evidence regarding SSBs and motor function is limited, as it only focuses on physical activity and sedentary time (7). Although research has predominantly reported that long durations of ST are associated with increased SSB intake (8–11), the relationship between PA and SSB intake is still debated. Ranjit et al. and Gan et al. reported that high PA was associated with more SSB intake (8, 11); however, a study conducted on African Americans showed that high PA was associated with both healthy and unhealthy diets (12), indicating that the findings regarding the relationship between motor function and SSB intake are inconsistent (7). Animal studies have also supported the association between SSBs and motor function (13), as excessive sugar intake was shown to activate the oxidative stress pathway in the human brain (13). During the key period of brain development, oxidative stress may affect the myelinization of white matter (14), thus potentially leading to motor function impairment. Both PA and ST could reflect motor competence, as individuals who lack motor skills are also less motivated to partake in PA and are more likely to be sedentary (15). In this case, it is necessary to directly clarify the relationship between SSBs and motor skills and motor competence.

The previous research regarding SSBs and motor function has focused on typically developed children, while there is a lack of evidence from children with developmental disorders. Autism, a developmental disorder that impairs social function, also impacts motor function in children, thus posing high risks to their physical health in their future life (16). In addition, children with autism tend to show peculiar food preferences, such as particular interests in consuming sugary or high-calorie food (17), which may also affect their SSB-intake behavior. Clarifying the relationship between motor function and SSB intake in children with autism could aid in providing a better description of the lifestyle of these children and in addressing the potential health risks imposed by this lifestyle. In this regard, we propose that it is worth exploring the association between SSBs and lifestyles in both ASD and TD children, to support a targeted intervention for SSB-related negative health outcomes. In this research, we explored the association between SSB intake and PA, ST, and the motor coordination function of children. Both typically developed children (who are described as possessing normally developed motor function) and children with autism (ASD) (who have partially impaired brain function) were included, in order to examine the association between SSBs and motor function in the overall child population. Considering the fact that executive function (EF) is impaired in children with autism (18), and that EF is related to both PA and motor coordination function (19), we employed EF as a covariate in the adjusted model. According to the literature review, we hypothesize that (a) children who consume more SSBs are more likely to adopt a low PA and high ST behavior pattern, and their motor competence will also be lower; (b) children with ASD exhibit lower PA, higher ST, and worse motor coordination function, compared with TD children, while increased SSB intake is associated with less PA and more ST, as well as lower motor coordination function, among both ASD and TD children.

## Methods

### Study Population and Procedure

Children with ASD (aged 6–9 years, *n* = 108) were obtained from an ongoing study, “The Guangzhou Longitudinal Study of Children with ASD,” examining the developmental trajectories of children with ASD in Guangzhou, China. These children were recruited between 2017 and 2021 from the Center for Child and Adolescent Psychology and Behavioral Development at Sun Yat-sen University. Each child had a historical diagnosis of ASD, which was further confirmed by a Childhood Autism Rating Scale (CARS) assessment, as well as an assessment conducted by two professional child psychiatrists in the research team (JJ and XL), using the Diagnostic and Statistical Manual of Mental Disorders-Fifth Edition (DSM-5). Simultaneously, age- and gender-matched typically developed children (TD, *n* = 207) were recruited *via* online media. We excluded children with a physical handicap as well as those with neurodevelopmental disorders other than ASD (e.g., attention-deficit/hyperactivity disorder, ADHD). Parent-reported questionnaires were employed to obtain information on the children, including demography, sugar-sweetened beverage intake, physical activity, sedentary time, motor coordination, and executive function. If one family had more than one child who fitted the inclusion criteria, we only included the eldest child. This study was approved by the Ethics Committee of Sun Yat-sen University and written informed consent was obtained from the parents of the child.

### Sugar-Sweetened Beverage Intake

Information regarding the sugar-sweetened beverage intake of the child was obtained by the question, “In the last 7 days, how many times did your child have sugar-sweetened beverages? Sugar-sweetened beverages included those beverages containing sugar, such as Coke, Sprite, bottled juice with sugar, Red Bull, etc. Please specify the exact number, and at each time, how many cups did your child have these sugar-sweetened beverages? One cup equals 250 ml.” The parents of the children were asked to specify the frequency and volume of SSB intake.

### Physical Activity and Sedentary Time

#### Physical Activity

Vigorous physical activity (VPA) was assessed by the question, “In the last 7 days, how many days did your child have vigorous physical activity? Vigorous physical activity means an activity that causes people to be out of breath, perspire and experience extreme exhaustion, such as basketball, football, carrying a heavy load, etc. Please specify the number of days. These days, how long did your child usually spend in these physical activities? Please specify the accurate time (hours and minutes).”

Moderate physical activity (MPA) was assessed by the question, “In the last 7 days, how many days did your child have moderate intensively physical activity? Moderate physical activity means an activity that causes people to mildly perspire and experience slight exhaustion, such as bicycling, playing table tennis, badminton, etc., but not including walking. Please specify the number of days. These days, how long did your child usually spend in these physical activities? Please specify the accurate time (hours and minutes).”

Walking was assessed by the question, “In the last 7 days, how many days did your child have walked (please only include and accumulate those walking that lasted 10 min or longer)? Walking includes those that happened at school, home, commute between school and home, and for exercise. Please specify the number of days. These days, how much time did your child usually spend in these walking? Please specify the accurate time (hours and minutes).”

#### Sedentary Time

Sedentary time was assessed by the question, “In the last 7 days, how long did your child have usually spend sitting or lying still each day (sedentary time includes sitting still at school, home, or other places, but does not include sleeping time)? Please specify the daily sedentary time and report the time of workdays (Monday to Friday) and holidays (Saturday and Sunday, other legal holidays) separately.”

### Motor Coordination Assessment

Motor coordination was assessed using the Developmental Coordination Disorder Questionnaire (DCDQ, Chinese version) (20), which is a 17-item parent-reported questionnaire consisting of three subscales that measure three domains of motor coordination, i.e., fine motor/handwriting, general coordination, and control during movement. This questionnaire provides a total score ranging from 17 to 85, as well as three subscale scores, with higher scores indicating better motor coordination function. The Chinese version of DCDQ covered the age range from kindergarten children to primary school-aged children (3–12 years) (21); it could be employed to assess, with acceptable reliability and validity, both typically developed children who have suspected motor coordination problems and children with neurodevelopmental disorders, such as ASD (22) or ADHD (23).

#### Potential Cofounders

The following characteristics were included as potential confounders: sociodemographic information, such as the child's gender, age, maternal/paternal age, maternal/paternal education, family's household income, and executive function. Sociodemographic information was collected using a parent-reported questionnaire and executive function was evaluated by the Behavior Rating Inventory of Executive Function (BRIEF) (24).

### Statistical Analysis

Data analyses were conducted in February 2022, and statistical analyses were performed using Statistic Package for Social Science 25.0 (SPSS 25.0, IMB, U.S., 2017). Continuous variables and categorical variables are presented as mean (standard deviation [*SD*]) values and percentages, respectively. The *T*-tests (for continuous variables) or chi-square tests (for categorical variables) were employed to explore the differences between ASD and TD children. A general linear model with several potential adjusted cofounders was used to evaluate the differences between ASD and TD children, and SSB intake was grouped and treated as one fixed factor, in order to test the potential interaction effect between SSB intake and ASD/TD group. All the tests employed were two-sided tests, and *p* < 0.05 was considered statistically significant.

## Results

A total of 107 ASD children and 209 TD children were included in the final analysis. The basic characteristics of both groups are described in Table 1. Compared with the TD group, there were a higher proportion of males in the ASD group (84.1 *vs*. 54.5%, ASD *vs*. TD, respectively, *p* < 0.001; Table 1). In addition, the ASD group had a lower maternal education level (college degree: 58.9 *vs*. 75.1%, ASD *vs*. TD, respectively, *p* = 0.003; Table 1) and a lower proportion of per capita family income over CNY 8000/month (43.0 *vs*. 74.6%, ASD *vs*. TD, respectively, *p* < 0.001; Table 1). We also observed a lower score on the executive function test among ASD children (54.08 ± 8.71 *vs*. 63.35 ± 9.16, ASD *vs*. TD, respectively, *p* < 0.001; Table 1), compared with TD children. No differences were observed in terms of child's age, maternal and paternal age, paternal education level, and SSB intake between the two groups (Table 1).

**Table 1:** Basic characteristics of autism spectrum disorder (ASD) children and typically developed (TD) children (*n* = 315).

	**ASD (*n* = 107)**	**TD (*n* = 208)**	***P*-value**
**Child characteristics**			
Age (mean ± SD)	7.71 ± 1.30	7.76 ± 1.32	0.745
**Gender** (%)			<0.001*
Boys	84.1%	54.5%	
Girls	15.9%	45.5%	
**Family characteristics**			
Maternal age (mean ± SD)	36.92 ± 3.42	37.06 ± 3.51	0.749
Paternal age (mean ± SD)	39.3 ± 4.7	39.2 ± 4.2	0.883
**Maternal college degree** (%)			0.003*
No	41.1%	24.9%	
Yes	58.9%	75.1%	
Paternal education (%)			0.752
Junior college or below	37.4%	35.6%	
College or higher	62.6%	64.4%	
**Per capita monthly household income (%)**			<0.001*
Less than CNY 8,000/month	57.0%	25.4%	
More than CNY 8,000/month	43.0%	74.6%	
Executive Function	63.35 ± 9.16	54.08 ± 8.71	<0.001*
Sugar-Sweetened beverage intake (ml/week)	335.86 ± 40.36	300.57 ± 28.88	0.487
**Sugar-Sweetened beverage intake (%)**			0.591
No intake	36 (33.6)	83 (39.7)	
Small to medium intake	37 (34.6)	66 (31.6)	
High intake	34 (31.8)	60 (28.7)	

*ASD, autism spectrum disorder; TD, typically developed; SD, standard deviation*.

*P <0.05.

Compared with TD children, ASD children were shown to spend less time performing high-intensity physical activity each week (2.77 ± 0.49 h *vs*. 4.23 ± 0.34 h, ASD *vs*. TD, respectively, *p* = 0.015) and less time being sedentary on weekdays (3.67 ± 0.28 h *vs*. 5.52 ± 1.89 h, ASD *vs*. TD, respectively, *p* < 0.001), weekends (2.68 ± 0.24 h *vs*. 3.49 ± 0.16 h, ASD *vs*. TD, respectively, *p* = 0.005), and per week (23.69 ± 1.69 *vs*. 34.59 ± 1.15, ASD *vs*. TD, respectively, *p* < 0.001). In terms of motor coordination function, ASD children showed lower scores for both the total scale (56.33 ± 1.10 *vs*. 67.67 ± 0.75, ASD *vs*. TD, respectively, *p* < 0.001) and the three subdomains of the scale: fine motor/handwriting (12.30 ± 0.38 *vs*. 14.21 ± 0.26, ASD *vs*. TD, respectively, *p* < 0.001), general coordination (25.17 ± 0.53 *vs*. 28.90 ± 0.36, ASD *vs*. TD, respectively, *p* < 0.001), and control during movement (18.86 ± 0.53 *vs*. 24.56 ± 0.36, ASD *vs*. TD, respectively, *p* < 0.001). In addition, more children in the ASD group were classified as having motor coordination problems (abnormal: 47.3 *vs*. 15.4%, ASD *vs*. TD, respectively, *p* < 0.001). No statistical differences were observed in the time spent performing moderate- or low-intensity physical activity each week (Table 2, all *p* > 0.05), between ASD and TD children.

**Table 2:** Comparison of physical activity, sedentary time, and developmental coordination disorder (DCD) score [mean ± standard deviation (SD)] between ASD and TD children^a^.

	**ASD (*n* = 107)**	**TD (*n* = 208)**	***P-*value**
**Physical activity (h/per week)**			
Time of high-intensity physical activity	2.77 ± 0.49	4.23 ± 0.34	0.015*
Time of moderate-intensity physical activity	3.64 ± 0.42	3.96 ± 0.29	0.540
Time of low-intensity physical activity	4.24 ± 0.43	4.50 ± 0.31	0.522
**Sedentary time (h/per day)**			
Weekdays	3.67 ± 0.28	5.52 ± 1.89	<0.001*
Weekends	2.68 ± 0.24	3.49 ± 0.16	0.005*
Total (h/per week)	23.69 ± 1.69	34.59 ± 1.15	<0.001*
**DCD score**			
Fine motor and handwriting	12.30 ± 0.38	14.21 ± 0.26	<0.001*
General coordination	25.17 ± 0.53	28.90 ± 0.36	<0.001*
Control during movement	18.86 ± 0.53	24.56 ± 0.36	<0.001*
DCD Total score	56.33 ± 1.10	67.67 ± 0.75	<0.001*
**Motor coordination abnormal**			<0.001*
Yes	47.3%	15.4%	
No	52.7%	84.6%	

*ASD, autism spectrum disorder; TD, typically developed; DCD, developmental coordination disorder; SD, standard deviation*.

*^a^The model adjusted for gender, maternal education, and executive function.*

*P <0.05.

Stratified by SSB intake volume (Table 3), we found that, in TD children, there was a significant difference in sedentary time among the three SSB intake groups. TD children with small to medium SSB intake spent the least time being sedentary on weekdays (5.83 ± 0.28, 4.54 ± 0.31, and 6.09 ± 0.31—no intake, small to medium intake, and high intake, respectively; *p* < 0.05), weekends (3.57 ± 0.24, 2.9 ± 0.26, and 3.94 ± 0.27 – no intake, small to medium intake, and high intake, respectively; *p* < 0.05), and during the entire week (36.31 ± 1.68, 28.48 ± 1.87, and 38.33 ± 1.89—no intake, small to medium intake, and high intake, respectively; *p* < 0.05). In addition, TD children with small to medium SSB intake showed the lowest fine motor and handwriting scores (14.72 ± 0.39, 13.37 ± 0.43, and 14.38 ± 0.44—no intake, small to medium intake, and high intake, respectively; *p* < 0.05).

**Table 3:** Relationship of intake of SSBs (mean ± SD) to physical activity, sedentary lifestyle, DCD score, and sleeping status among ASD and TD children^#^.

	**ASD (*****n*** **=** **107)**			**TD (*****n*** **=** **208)**			***P*_group_**	***P*_intake_**	*P*_group*intake_
	**No intake (*n* = 36)**	**Small to medium intake (*n* = 37)**	**High intake (*n* = 34)**	**No intake (*n* = 83)**	**Small to medium intake (*n* = 66)**	**High intake (*n* = 59)**			
**Physical activity (h/per week)**									
VPA	2.86 ± 0.75^a^	3.02 ± 0.74^a^	2.27 ± 0.81^a^	4.53 ± 0.5^a^	4.15 ± 0.56^a^	3.97 ± 0.57^a^	0.013	0.639	0.875
MPA	4.08 ± 0.65^a^	3.12 ± 0.64^a^	3.78 ± 0.7^a^	3.86 ± 0.44^a^	4.09 ± 0.48^a^	3.92 ± 0.49^a^	0.567	0.792	0.521
Walking	3.48 ± 0.78^a^	4.56 ± 0.77^a^	4.47 ± 0.84^a^	5 ± 0.52^a^	4.17 ± 0.58^a^	4.48 ± 0.59^a^	0.544	0.937	0.293
**Sedentary time**			
Week days (h/per day)	3.86 ± 0.42^a^	3.60 ± 0.41^a^	3.41 ± 0.45^a^	5.83 ± 0.28^a^	4.54 ± 0.31^b^	6.09 ± 0.31^a^	<0.001	0.048	0.044
Weekends (h/per day)	3.14 ± 0.35^a^	2.27 ± 0.35^a^	2.62 ± 0.38^a^	3.57 ± 0.24^a^	2.9 ± 0.26^b^	3.94 ± 0.27^a^	0.005	0.015	0.302
Total (h/per week)	25.57 ± 2.52^a^	22.53 ± 2.49^a^	22.29 ± 2.7^a^	36.31 ± 1.68^a^	28.48 ± 1.87^b^	38.33 ± 1.89^a^	<0.001	0.017	0.062
**DCD score**			
Fine motor and handwriting	13.03 ± 0.58^a^	11.66 ± 0.57^a^	12.06 ± 0.62^a^	14.72 ± 0.39^a^	13.37 ± 0.43^b^	14.38 ± 0.44^a^	<0.001	0.016	0.770
General coordination	25.25 ± 0.82^a^	25.43 ± 0.80^a^	24.69 ± 0.88^a^	29.17 ± 0.54^a^	29.1 ± 0.61^a^	28.39 ± 0.62^a^	<0.001	0.517	0.979
Control during movement	18.93 ± 0.81^a^	18.91 ± 0.80^a^	18.67 ± 0.87^a^	24.56 ± 0.54a	23.78 ± 0.6a	25.34 ± 0.62a	<0.001	0.631	0.427
DCD total score	57.21 ± 1.67^a^	56 ± 1.65^a^	55.42 ± 1.8^a^	68.45 ± 1.12^a^	66.25 ± 1.24^a^	68.11 ± 1.28^a^	<0.001	0.456	0.691
DCD (abnormal%)	38.6%^a^	51.4%^a^	55.0%^a^	13.8%^a^	17.0%^a^	15.7%^a^	<0.001	0.451	0.786

*VPA, vigorous physical activity; MPA, moderate physical activity; DCD, developmental coordination disorder; ASD, autism spectrum disorder, TD, typically developed; SD, standard deviation*.

*^#^The model adjusts for gender, maternal education, and executive function.*

*^ab^Data with different upper letters had statistically significant differences in the pairwise comparisons (P <0.05).*

*P <0.0.5.

We observed an interaction effect between groups and SSB intake in terms of weekday sedentary time (*p* = 0.044, Table 3). In this case, a simple effect has been tested. In the ASD group, the weekday sedentary time remained relatively stable among children with different SSB intake levels (3.86 ± 0.42, 3.60 ± 0.41, and 3.41 ± 0.45—no intake, small to medium intake, and high intake, respectively; *p* > 0.05; Table 3), while the children with small to medium SSB intake in the TD group showed the lowest weekday sedentary time (5.83 ± 0.28, 4.54 ± 0.31, and 6.09 ± 0.31—no intake, small to medium intake, and high intake, respectively; *p* < 0.05; Table 3). No significant interactions between physical activity and DCD score were observed (Table 3, all *p* > 0.05).

## Discussion

This study aimed to gain insights into SSB intake and motor function among autistic and typically developed children. Firstly, we observed a relatively lower vigorous PA time, overall sedentary time, and motor coordination function among ASD children, relative to TD children. Secondly, TD children with small to medium SSB intake showed less sedentary time and lower motor coordination function, relative to TD children with no SSB intake or high SSB intake. However, children with ASD did not exhibit the same trend.

This research is significant and possesses many strengths; it provides (1) new information regarding the association between SSBs and brain health, especially motor coordination function among children with or without neural disorder (autism in this case), (2) an improved understanding of SSBs and the risks they may pose to a child's health, and, thus, (3) a potential intervention target for assessing motor function in school-aged children.

Research that has addressed the accelerometer-measured PA of ASD children has predominately shown that it is lower relative to TD children (25), while we only showed lower VPA among ASD children. This could be attributed to the parent-reported assessment of PA in our study. Relatively lower VPA could represent a neural basis for the social difficulties experienced by ASD children (26), as most VPA requires cooperation with peers and strong volition to complete; however, ASD children lack both these characteristics. Therefore, children with ASD could experience great difficulty in performing VPA. Low motor competence in ASD children has been widely observed in the previous research (27), as well as in our study (Table 2). The lack of necessary motor skills could directly impede the ability of a child with ASD to partake in physical activity, which is also a potential reason for the decreased time spent performing PA. In the previous research, the sedentary time of ASD children has been debated, as some researchers reported no significant difference in ST between ASD and TD children (28, 29), while other researchers reported more ST among ASD children than TD children (30). However, these researchers failed to consider the potential repetitive symptoms of ASD, which are widely observed among ASD children (31). Repetitive behavior includes aimless wandering, turning around, and other behaviors involving activity (31) that are not considered to be PA or ST. Thus, ASD children could show less PA as well as ST.

The abundance of fine sugar contained in SSBs was concluded to be the reason for the rapid increase in blood glucose. It may explain the *U*-shape in the association between SSB intake and ST. Relatively high glucose levels are proven to be linked with low (32) - grade inflammation in humans (33), the latter of which could cause fatigue (34); consequently, an individual with relatively high glucose levels shows less active and more sedentary behavior. On the other hand, relatively low glucose levels may have a similar effect, as bodily activity is energy supplementary dependent; therefore, low glucose levels can hardly support the activity of the human body.

In TD children, those with small to medium SSB intake or high SSB intake showed relatively lower scores in fine motor and handwriting skills, which could support the SSB–brain function hypothesis. The relationship between motor coordination function and SSBs has been confirmed by an animal study, and the causation was attributed to oxidative stress originating from excessive SSB intake (35). Motor skills, particularly fine motor and handwriting skills, require white matter integrity during the child's cerebrum–cerebellum development (36), while excessive fine sugar intake affects the internal environment of white matter myelination, by increasing the level of inflammation (33). On the other hand, regardless of the fine sugar present in SSBs, caffeine and additives could be bioactive and damage motor function (37), which is potentially related to fine motor and handwriting skills.

A study in the U.S.A. showed that individuals with autism had a low-quality diet and high-SSB intake, compared with nationally representative data (38). In addition, ASD children had unique food preferences (39), which may have also affected their diet, particularly SSB intake. The previous research reported that individuals with the developmental disabilities were more susceptible to the negative health outcomes associated with SSBs (40); however, this also depends on the education level of the child's mother. A relatively highly educated mother may decrease their child's SSB intake (41), therefore, reducing the associated risks, considering the fact that over 50% of the mothers in the ASD group had a bachelor's degree or above, which may explain the lack of differences in SSB intake between ASD and TD children in our study.

This research has a few limitations. Firstly, the data collection was conducted using parent-reported questionnaires, which compromised the reliability. Secondly, the relatively small sample size in the ASD group also limited the statistical power. Moreover, we only included sugar-sweetened beverages, but the consumption of other sweet products also provided sugar in the children's daily diets; thus, the sugar intake in our study is underestimated. The recruitment of TD children *via* online media may have affected the representativity of the control group. It is also worth mentioning that the relatively low intake of SSBs in the research population decreased discrimination among the groups; this may affect the results of this research. A population with higher average SSB intake should be identified in future studies, to confirm our findings. In addition, the cross-sectional design of the study means that the causation can hardly be tested. Future research should focus on a longitudinally designed, large sample-sized study, as well as objective measurements of activity.

## Conclusion

In this research, we described the PA, ST, and motor coordination function of both typically developed children and children with autism, and confirmed the relatively low PA and low ST lifestyles in children with autism. The association between SSBs and motor function in typically developed children is also addressed. The results showed the necessity of supporting children with lifestyle interventions and promoting early lifestyle interventions, therefore, decreasing the risk of long-term health problems.

This study also provided important evidence showing that children with special conditions should be supported with lifestyle interventions, including the promotion of motor function training to encourage a healthier lifestyle.

## Data Availability Statement

The datasets used and/or analyzed during the current study are available from the corresponding author on reasonable request.

## Ethics Statement

The studies involving human participants were reviewed and approved by the Ethical Review Committee for Biomedical Research, Sun Yat-sen University. Written informed consent to participate in this study was provided by the participants' legal guardian/next of kin.

## Author Contributions

Concept and design and drafting of the manuscript: MC. Acquisition, analysis, or interpretation of data: TG, CJ, and MC. Critical revision of the manuscript for important intellectual content: XL and JJ. Statistical analysis: TG. Obtained funding: JJ. All authors contributed to the article and approved the submitted version.

## Funding

This study was supported by the Key-Area Research and Development Program of Guangdong Province (Grant No. 2019B030335001), the National Science Foundation of China (Grant Nos: 81872639 and 81903337), and National Social Science Foundation of China (20&ZD296).

## Conflict of Interest

The authors declare that the research was conducted in the absence of any commercial or financial relationships that could be construed as a potential conflict of interest.

## Publisher's Note

All claims expressed in this article are solely those of the authors and do not necessarily represent those of their affiliated organizations, or those of the publisher, the editors and the reviewers. Any product that may be evaluated in this article, or claim that may be made by its manufacturer, is not guaranteed or endorsed by the publisher.

## References

[1] Poorolajal J Sahraei F Mohamdadi Y Doosti-Irani A Moradi L. Behavioral factors influencing childhood obesity: a systematic review and meta-analysis. Obes Res Clin Pract. (2020) 14:109–18. 10.1016/j.orcp.2020.03.002 32199860

[2] Noble EE Olson CA Davis E Tsan L Chen Y Schade R. Gut microbial taxa elevated by dietary sugar disrupt memory function. Transl Psychiat. (2021) 11:194. 10.1038/s41398-021-01309-7 33790226 PMC8012713

[3] Kendig MD. Cognitive and behavioural effects of sugar consumption in rodents. A review. Appetite. (2014) 80:41–54. 10.1016/j.appet.2014.04.028 24816323

[4] Anjum I Jaffery SS Fayyaz M Wajid A Ans AH. Sugar beverages and dietary sodas impact on brain health: a mini literature review. Cureus. (2018) 10:e2756. 10.7759/cureus.2756 30094113 PMC6080735

[5] Cohen JFW Gorski MT Gruber SA Kurdziel LBF Rimm EB. The effect of healthy dietary consumption on executive cognitive functioning in children and adolescents: a systematic review. Brit J Nutr. (2016) 116:989–1000. 10.1017/S0007114516002877 27487986

[6] Reichelt AC Killcross S Hambly LD Morris MJ Westbrook RF. Impact of adolescent sucrose access on cognitive control, recognition memory, and parvalbumin immunoreactivity. Learn Mem. (2015) 22:215–24. 10.1101/lm.038000.114 25776039 PMC4371171

[7] Marinoni M Parpinel M Gasparini A Ferraroni M Edefonti V. Risky behaviors, substance use, and other lifestyle correlates of energy drink consumption in children and adolescents: a systematic review. Eur J Pediatr. (2022) 181:1307–19. 10.1007/s00431-021-04322-6 34988663

[8] Ranjit N Evans MH Byrd-Williams C Evans AE Hoelscher DM. Dietary and activity correlates of sugar-sweetened beverage consumption among adolescents. Pediatrics. (2010) 126:e754–61. 10.1542/peds.2010-1229 20876172 PMC3045775

[9] Barrett P Imamura F Brage S Griffin SJ Wareham NJ Forouhi NG. Sociodemographic, lifestyle and behavioural factors associated with consumption of sweetened beverages among adults in cambridgeshire, uk: the fenland study. Public Health Nutr. (2017) 20:2766–77. 10.1017/S136898001700177X 28789721 PMC6020996

[10] Fletcher EA McNaughton SA Crawford D Cleland V Della Gatta J Hatt J. Associations between sedentary behaviours and dietary intakes among adolescents. Public Health Nutr. (2018) 21:1115–22. 10.1017/S136898001700372X 29317000 PMC5848750

[11] Gan WY Mohamed SF Law LS. Unhealthy lifestyle associated with higher intake of sugar-sweetened beverages among Malaysian school-aged adolescents. Int J Env Res Pub He. (2019) 16:2785. 10.3390/ijerph16152785 31382672 PMC6696103

[12] Maher JP Harduk M Hevel DJ Adams WM McGuirt JT. Momentary physical activity co-occurs with healthy and unhealthy dietary intake in african american college freshmen. Nutrients. (2020) 12:1360. 10.3390/nu12051360 32397433 PMC7285035

[13] Poulose SM Miller MG Scott T Shukitt-Hale B. Nutritional factors affecting adult neurogenesis and cognitive function. Adv Nutr Int Rev J. (2017) 8:804–11. 10.3945/an.117.016261 29141966 PMC5683005

[14] Spaas J van Veggel L Schepers M Tiane A van Horssen J Wilson DM. Oxidative stress and impaired oligodendrocyte precursor cell differentiation in neurological disorders. Cell Mol Life Sci. (2021) 78:4615–37. 10.1007/s00018-021-03802-0 33751149 PMC8195802

[15] King-Dowling S Proudfoot NA Cairney J Timmons BW. Motor competence, physical activity, and fitness across early childhood. Med Sci Sports Exerc. (2020) 52:2342–8. 10.1249/MSS.0000000000002388 32366800

[16] Bhat AN. Motor impairment increases in children with autism spectrum disorder as a function of social communication, cognitive and functional impairment, repetitive behavior severity, and comorbid diagnoses: a spark study report. Autism Res. (2021) 14:202–19. 10.1002/aur.2453 33300285 PMC8176850

[17] Chistol LT Bandini LG Must A Phillips S Cermak SA Curtin C. Sensory sensitivity and food selectivity in children with autism spectrum disorder. J Autism Dev Disord. (2018) 48:583–91. 10.1007/s10803-017-3340-9 29116421 PMC6215327

[18] Ellis WS Kaushanskaya M Larson C Mathee J Bolt D. Executive function skills in school-age children with autism spectrum disorder: association with language abilities. J Speech Lang Hear Res. (2018) 61:2641–58. 10.1044/2018_JSLHR-L-RSAUT-18-0026 30418493 PMC6693571

[19] de Greeff JW Bosker RJ Oosterlaan J Visscher C Hartman E. Effects of physical activity on executive functions, attention and academic performance in preadolescent children: a meta-analysis. J Sci Med Sport. (2018) 21:501–7. 10.1016/j.jsams.2017.09.595 29054748

[20] Wilson BN Kaplan BJ Crawford SG Campbell A Dewey D. Reliability and validity of a parent questionnaire on childhood motor skills. Am J Occup Ther. (2000) 5:484–93. 10.5014/ajot.54.5.484 11006808

[21] HuaJDuWDaiXWuMCaiXShenM. International clinical practice recommendations on the definition, diagnosis, assessment, intervention, and psychosocial aspects of developmental coordination disorder—Chinese (Mandarin) translation. Dev Med Child Neurol. (2020) 61:242–85. 10.1111/dmcn.1469333249559

[22] Bhat AN. Is motor impairment in autism spectrum disorder distinct from developmental coordination disorder? A report from the spark study. Phys Ther. (2020) 100:633–44. 10.1093/ptj/pzz190 32154876 PMC7297441

[23] Lee J Mayall LA Bates KE Hill EL Leonard HC Farran EK. The relationship between motor milestone achievement and childhood motor deficits in children with attention deficit hyperactivity disorder (adhd) and children with developmental coordination disorder. Res Dev Disabil. (2021) 113:103920. 10.1016/j.ridd.2021.103920 33845359

[24] Baron IS. Behavior rating inventory of executive function. Child Neuropsychol. (2000) 6:235–8. 10.1076/chin.6.3.235.3152 11419452

[25] Liang X Li R Wong SHS Sum RKW Sit CHP. Accelerometer-measured physical activity levels in children and adolescents with autism spectrum disorder: a systematic review. Prev Med Rep. (2020) 19:101147. 10.1016/j.pmedr.2020.101147 32637302 PMC7327848

[26] Craig F Crippa A Ruggiero M Rizzato V Russo L Fanizza I. Characterization of autism spectrum disorder (asd) subtypes based on the relationship between motor skills and social communication abilities. Hum Movement Sci. (2021) 77:102802. 10.1016/j.humov.2021.102802 33894651

[27] Downs SJ Boddy LM McGrane B Rudd JR Melville CA Foweather L. Motor competence assessments for children with intellectual disabilities and/or autism: a systematic review. BMJ Open Sport Exerc Med. (2020) 6:e902. 10.1136/bmjsem-2020-000902 33324486 PMC7722274

[28] Corvey K Menear KS Preskitt J Goldfarb S Menachemi N. Obesity, physical activity and sedentary behaviors in children with an autism spectrum disorder. Matern Child Health J. (2016) 20:466–76. 10.1007/s10995-015-1844-5 26515467

[29] McCoy SM Jakicic JM Gibbs BB. Comparison of obesity, physical activity, and sedentary behaviors between adolescents with autism spectrum disorders and without. J Autism Dev Disord. (2016) 46:2317–26. 10.1007/s10803-016-2762-0 26936162

[30] Martinez-Cayuelas E Rodriguez-Morilla B Soriano-Guillen L Merino-Andreu M Moreno-Vinues B Gavela-Perez T. Sleep problems and circadian functioning in children and adolescents with autism spectrum disorder. Pediatr Neurol. (2022) 126:57–64. 10.1016/j.pediatrneurol.2021.09.009 34740134

[31] Hyman SL Levy SE Myers SM. Executive summary: identification, evaluation, and management of children with autism spectrum disorder. Pediatrics. (2020) 145:e20193448. 10.1542/9781610024716-part01-ch002 31843858

[32] Bourre JM. Effects of nutrients (in food) on the structure and function of the nervous system: update on dietary requirements for brain. Part 2: macronutrients. J Nutr Health Aging. (2006) 10:386–99. 17066210

[33] Calder PC. Dietary factors and low grade inflammation in relation to overweight and obesity revisted. Br J Nutr. (2022) 127:1–9. 10.1017/S0007114522000782 35260214 PMC9044216

[34] Lacourt TE Vichaya EG Chiu GS Dantzer R Heijnen CJ. The high costs of low-grade inflammation: persistent fatigue as a consequence of reduced cellular-energy availability and non-adaptive energy expenditure. Front Behav Neurosci. (2018) 12:78. 10.3389/fnbeh.2018.00078 29755330 PMC5932180

[35] Khoshnoud MJ Siavashpour A Bakhshizadeh M Rashedinia M. Effects of sodium benzoate, a commonly used food preservative, on learning, memory, and oxidative stress in brain of mice. J Biochem Mol Toxic. (2018) 32:e22022. 10.1002/jbt.22022 29243862

[36] Franki I Mailleux L Emsell L Peedima M Fehrenbach A Feys H. The relationship between neuroimaging and motor outcome in children with cerebral palsy: a systematic review—part a. structural imaging. Res Dev Disabil. (2020) 100:103606. 10.1016/j.ridd.2020.103606 32192951

[37] Trasande L Shaffer RM Sathyanarayana S. Food additives and child health. Pediatrics. (2018) 142:e20181410. 10.1542/peds.2018-1410 30037972 PMC6298598

[38] Buro AW Gray HL Kirby RS Berkman K Agazzi H Shaffer-Hudkins E. Diet quality in an ethnically diverse sample of children and adolescents with autism spectrum disorder compared with nationally representative data. Disabil Health J. (2021) 14:100981. 10.1016/j.dhjo.2020.100981 32811783

[39] Peretti S Mariano M Mazzocchetti C Mazza M Pino MC Verrotti DPA. Diet: the keystone of autism spectrum disorder? Nutr Neurosci. (2019) 22:825–39. 10.1080/1028415X.2018.1464819 29669486

[40] Kim S Park S Carroll DD Okoro CA. Daily sugar-sweetened beverage consumption, by disability status, among adults in 23 states and the district of columbia. Prev Chronic Dis. (2017) 14:E132. 10.5888/pcd14.160606 29240555 PMC5737978

[41] Inhulsen MM Merelle SY Renders CM. Parental feeding styles, young children's fruit, vegetable, water and sugar-sweetened beverage consumption, and the moderating role of maternal education and ethnic background. Public Health Nutr. (2017) 20:2124–33. 10.1017/S1368980017001409 28712381 PMC10261429

